# Supplementary material for: Performance Assessment of Portable Optical Particle Spectrometer (POPS)
Source: Sensors (Basel). 2020 Nov 5;20(21):6294. doi: 10.3390/s20216294 (PMC7663837; doi:10.3390/s20216294)
Supplement: Supplementary file 1 [file sensors-20-06294-s001.pdf]

sensors

MDPI

Article

Performance Assessment of Portable Optical Particle Spectrometer (POPS)

Fan Mei [1,*], Gavin McMeeking [2], Mikhail Pekour [1,*], Ru-Shan Gao [3], Gourihar Kulkarni [1], Swarup China [1], Hagen Telg [4], Darielle Dexheimer [5], Jason Tomlinson [1] and Beat Schmid [1]

1 Pacific Northwest National Laboratory, Richland, WA, USA; mikhail.pekour@pnnl.gov (M.P.); Gourihar.Kulkarni@pnnl.gov (G.K.); swarup.china@pnnl.gov (S.C.); jason.tomlinson@pnnl.gov (J.T.); Beat.Schmid@pnnl.gov (B.S.)
2 Handix Scientific LLC, Boulder, CO, USA; gavin@handixscientific.com
3 NOAA Earth System Research Laboratory, Chemical Sciences Division, Boulder, CO, USA; RuShan.Gao@noaa.gov
4 Cooperative Institute for Research in Environmental Sciences (CIRES), University of Colorado, Boulder, CO, USA; hagen.telg@noaa.gov
5 Sandia National Laboratories, Albuquerque, NM, USA; ddexhei@sandia.gov
* Correspondence: fan.mei@pnnl.gov (F.M.); Mikhail.Pekour@pnnl.gov (M.P.); Tel.: +001-509-375-3965 (WA)

Received: 29 September 2020; Accepted: 30 October 2020; Published: date

Manufacturer Calibration

The manufacturer calibration set up consists of an atomizer connected to a desiccant dryer filled with silica gel, ^{210}Po aerosol neutralizer (Brechtel Manufacturing, Hayward, CA, USA), and the inlet of a TSI 3081 differential mobility analyzer. The DMA operates with a sheath flow of 5 LPM and a monodisperse flow of approximately 0.48 LPM, for an approximately 10:1 sheath-monodisperse flow ratio. The output of the DMA is split using a "Y" to the WCPC and POPS instrument under test. The DMA is configured to scan from approximately 100 nm to 500 nm over five minutes. The POPS and CPC record particle concentration, and the POPS also records particle the concentration of particles as a function of diameter. Concentrations are compared to produce a counting efficiency curve, which is used to determine the D_{50} (diameter where the POPS detects 50% of the input particles).

Particle Morphology and Refractive index Effects on POPS and PCASP

In this study, we used three test aerosols with a wide variety of refractive indices and morphologies (see Table 1); the PCASP was used as a reference sensor. Note that the PCASP has a passive flow inlet designed to work in flight mounted on aircraft under-wing pylon, which may lead to significant particle loss during the lab operation. The test aerosol of 500 nm was created and conditioned using the same apparatus as for counting efficiency study (Figure 1). It should be noted that the chosen diameter falls between wavelengths of the sensors' lasers: 405 nm and 632.8 nm. This diameter of 500 nm is far enough from the roll off of POPS's counting efficiency curve at the lower end, but still well below the area where Mie theory predicts oscillations of the scattered signal for most common aerosols (refractive index below 2).

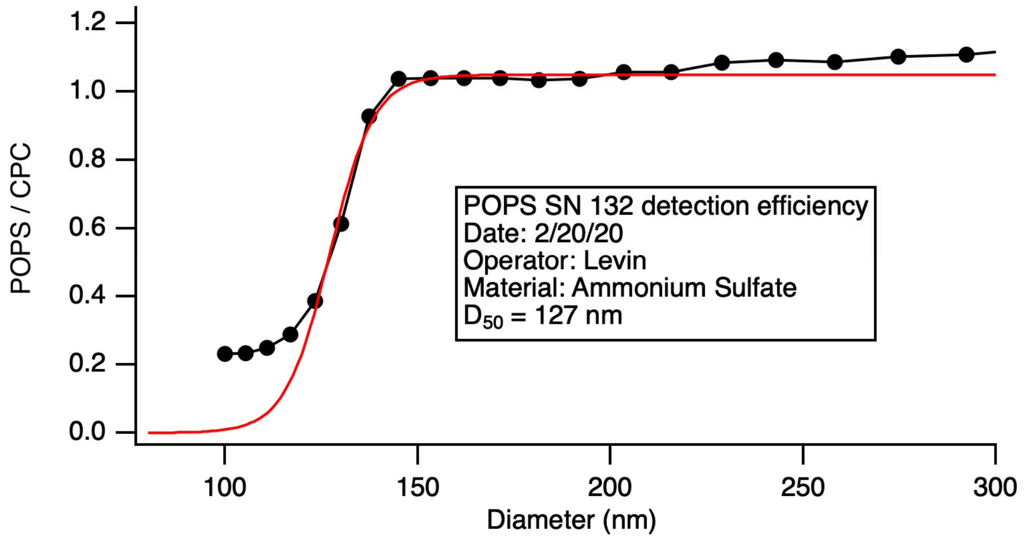

Figure 1. Manufacture example of the counting efficiency of POPS.

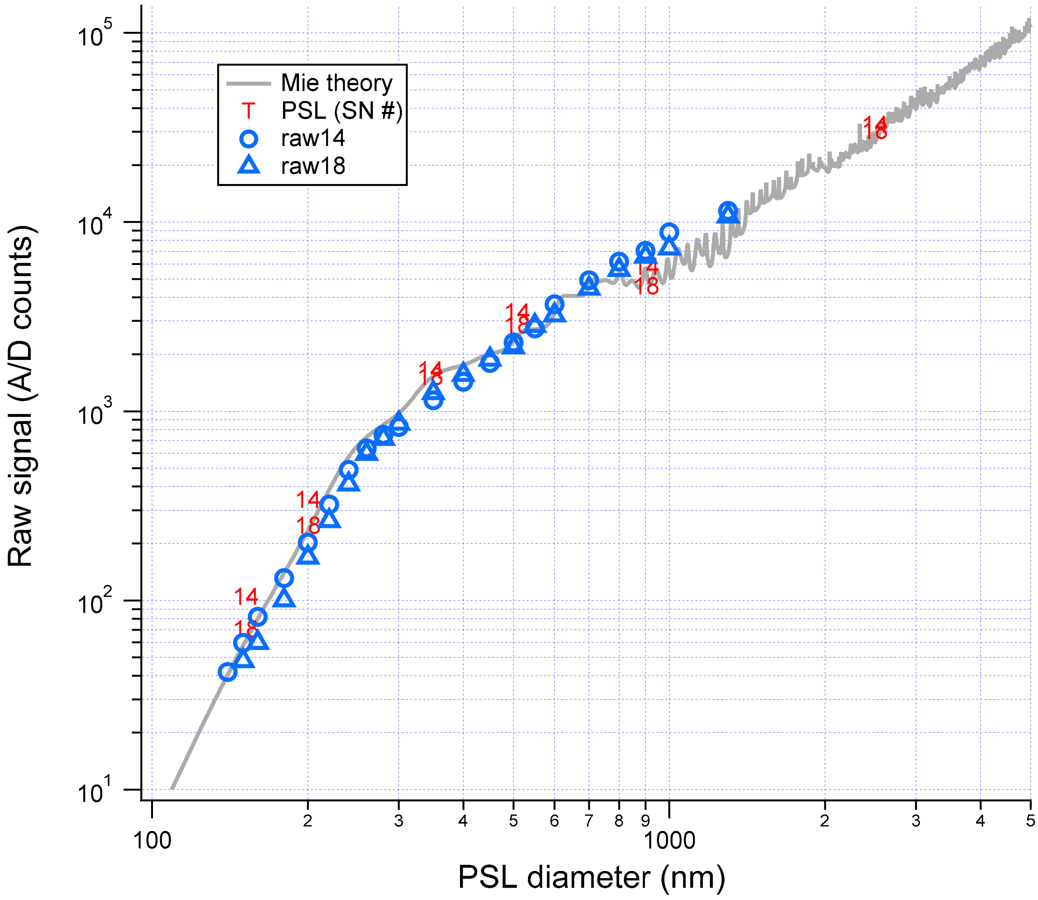

Figure 2. Calibration data of PSL particles for the other two POPS tested in this study.

Table 1. Comparison of measured aerosol concentrations (N in cm-3) from POPS, SMPS, and UHSAS.

N_{SMPS} (50–700 nm)	N_{UHSAS} (60–700 nm)	N_{POPS} (115–700 nm)	N_{POPS} (135–700 nm)	Std. of Baseline
19 ± 6.0	17 ± 6	184 ± 4	14 ± 4	8.79 ± 0.39

52 ± 11	54 ± 8	217 ± 4	47 ± 4	8.75 ± 0.39
699 ± 43	673 ± 50	768 ± 14	605 ± 14	8.62 ± 0.37
1892 ± 28	1702 ± 46	1931 ± 24	1755 ± 24	8.78 ± 0.41
4307 ± 22	3057 ± 59	3154 ± 46	3334 ± 46	8.97 ± 0.45
5472 ± 56	3814 ± 56	4472 ± 36	4278 ± 36	8.84 ± 0.41
14940 ± 1601	7204 ± 80	6668 ± 43	6489 ± 41	10.58 ± 0.83
15033 ± 1089	7840 ± 86	8443 ± 83	8241 ± 83	9.52 ± 0.51

Table 2. The RH effect on the aerosol the primary peak of the measured size distribution from POPS, SMPS, and UHSAS (using the 200 nm size-selected ammonium sulfate particles).

RH, %	Dp-pops, nm	Dp-UHSAS, nm	Dp-SMPS, nm
40 ≤ RH <71.5	185	192.4	201.7
71.5 ≤ RH <80.5	195	192.4 -> 221.8	201.7 -> 259.5
80.5 ≤ RH <91.7	210 -> 225	221.8 -> 234.7	289 -> 310.6
91.7 ≥ RH > 64.5	225 -> 210	234.7 -> 197.9	310.6 -> 259.5
64.5 ≥ RH > 42	195	197.9	259.5
42 ≥ RH	185	192.4	201.7

Morphology of the irregular shaped particles (ATD and TiO2) was examined with a Scanning Electron Microscope; examples of powder images are presented in Figure S5b and Figure S6b.

Figure S3–S6 show examples of size distributions measured by POPS and PCASP for 500 nm (electrical mobility size) test aerosols: ammonium sulfate, silicon dioxide, ATD, and TiO2, respectively. A secondary peak at the larger sizes on the distributions is the usual artifact of electrical mobility based separation systems; it is formed by the multiple charged particles passed through the DMA column [1].

In all cases, POPS showed the presence of particles in the first bin. This artifact was previously observed by the manufacturer, especially on units with marginal laser profile quality. The artifact is probably caused by false positive detection associated with oscillations in the baseline signals near "true" particle events. The developer (NOAA) and the manufacturer (Handix Scientific) have implemented the special procedure in the updated firmware to minimize the false detections, with more recently manufactured units displaying few if any false particle counts near the lower detection limit. However, despite these improvements, readings from the lowest bins should be interpreted with care; monitoring of the baseline and its standard deviation is advised.

The two instruments (the POSP and the PCASP) observed similar size distributions of ammonium sulfate aerosols consistent with the expected output of the DMA, which provides validation to the correctness of calibration of both optical instruments (Figure S3).

The tests with the three aerosols of irregular shape (Figure S4–S6) showed significant differences in POPS and PCASP responses. Several factors could have contributed to these discrepancies. Differentiation of irregularly shaped aerosols with a DMA often does not produce particles of desired size (e. g. equivalent volume diameter), yields broader size distributions than expected from used sample to sheath flow ratio, and produce a significant number of particles larger then DMA bandwidth [2]. Arizona Test Dust is a mixture of several mineral species of different physical properties (notably refractive index, shape factor, and density); it is usually characterized by some "effective" set of parameters. For example the value of complex refractive index we used is 1.51 + 0.001i (Table 1); however single particle in the mixture may have refractive index from 1.413 + 0.000773i (illite) to 3.102 + 0.0925i (hematite) [3]. Particle dynamic shape factor for ATD may vary in a wide range from 1.3 to 3.1 [2], which directly affects particle size selected by DMA, since equivalent electrical mobility diameter depends inversely on the dynamic shape factor, see Equation (1) [4]. All these processes contribute to the "smearing" of the measured size distributions.

Size distribution for the TiO_2 test, shown in Figure S6, has a rather sharp secondary peak between 200 and 300 nm, which can be explained by the complex morphology of TiO_2 particles. Sample batch

of TiO_2 "powder" we used seems to be a mixture of dendrite clusters of small size spherical particles (nominal diameter d_n of 21 nm) and larger, monolithic or collapsed dendrite particles (see Figure S6b). Titanium dioxide clusters can be described as fractal aggregates with fractal dimension D_f of 1.8 ([5]. Linear size of a fractal aggregate is traditionally described with a radius of gyration R_g; it was empirically found that in the case of aggregate consisting of a large number of monomers radius of gyration (R_g) can be related to electrical mobility diameter D_m as $D_m=1.4R_g$. [5] The relation between mass parameter (number of monomers in the aggregate N) and radius of gyration R_g can be described as: [6]

$$N = k_0 \left(\frac{R_g}{a_n}\right)^{D_f} \quad (1)$$

where k_0 is a constant in the order of unity (usually 1.3), $a_n=d_n/2$ is a radius of monomer sphere (in our case 10.5 nm). Equivalent volume diameter D_v can be expressed as $D_v = d_n N^{\frac{1}{3}}$. For particles with a nominal mobility diameter of 500 ± 83 nm, we have an equivalent volume diameter of $190 {}^{+18}_{-20}$ nm with some monomers in clusters peaked at 743 nm. A rather high value of the refractive index for TiO2 particles (2.682) resulted in an apparent shift of the peak to the right (Figure S6).

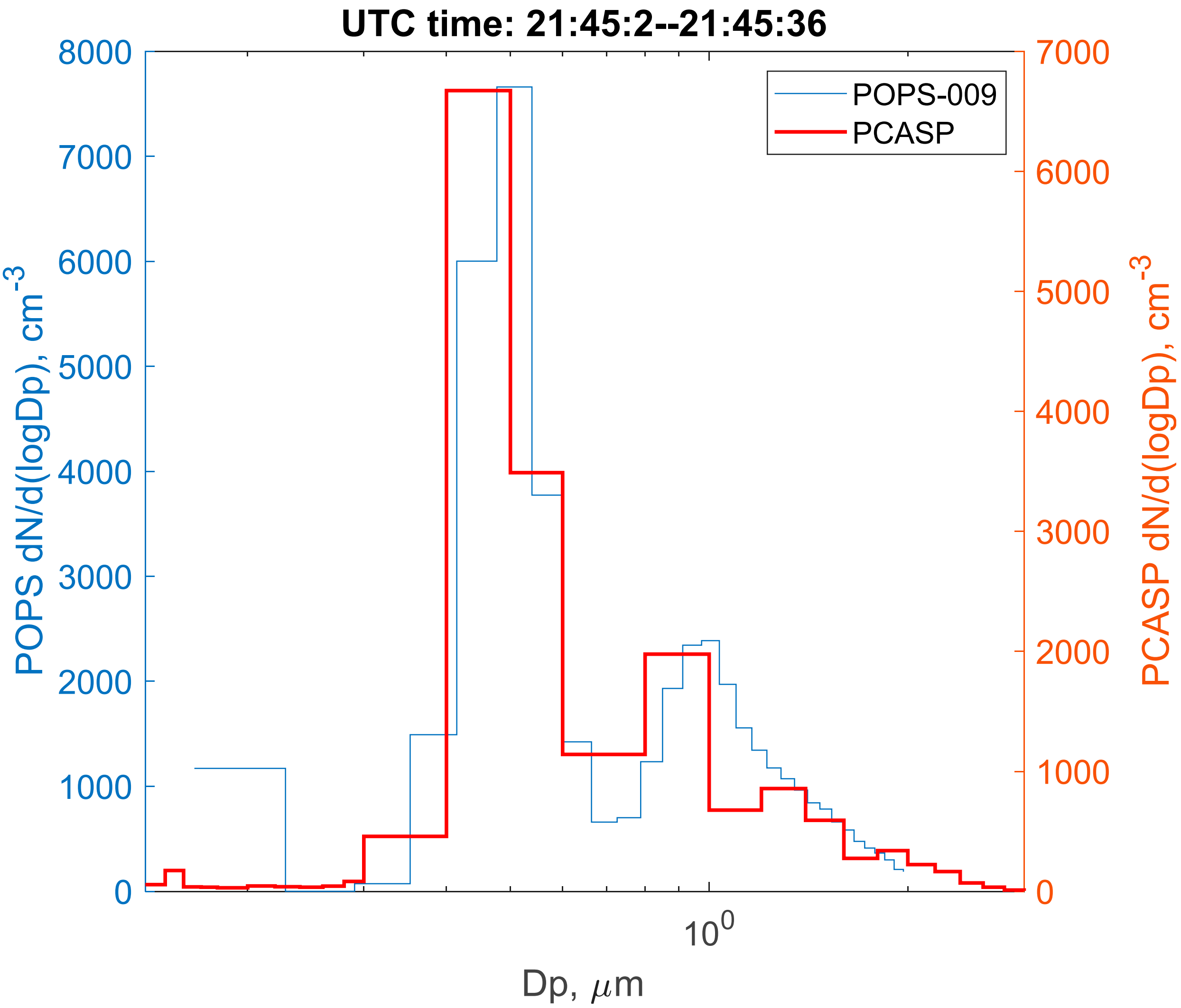

Figure S3. POPS and PCASP size distribution of 500 nm (electrical mobility size) ammonium sulfate particles.

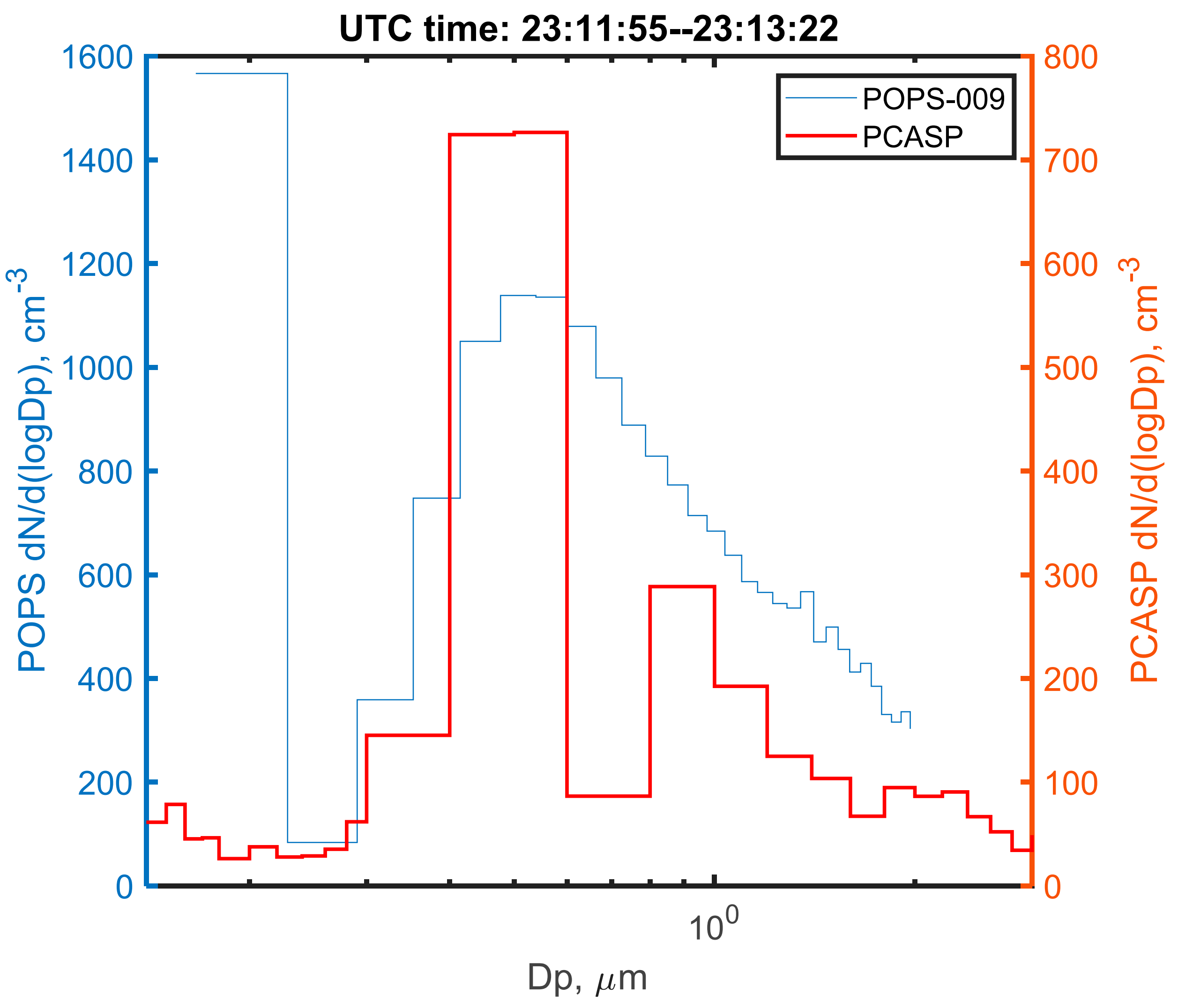

Figure S4. POPS and PCASP size distribution of 500 nm (electrical mobility size) silicon dioxide particles.

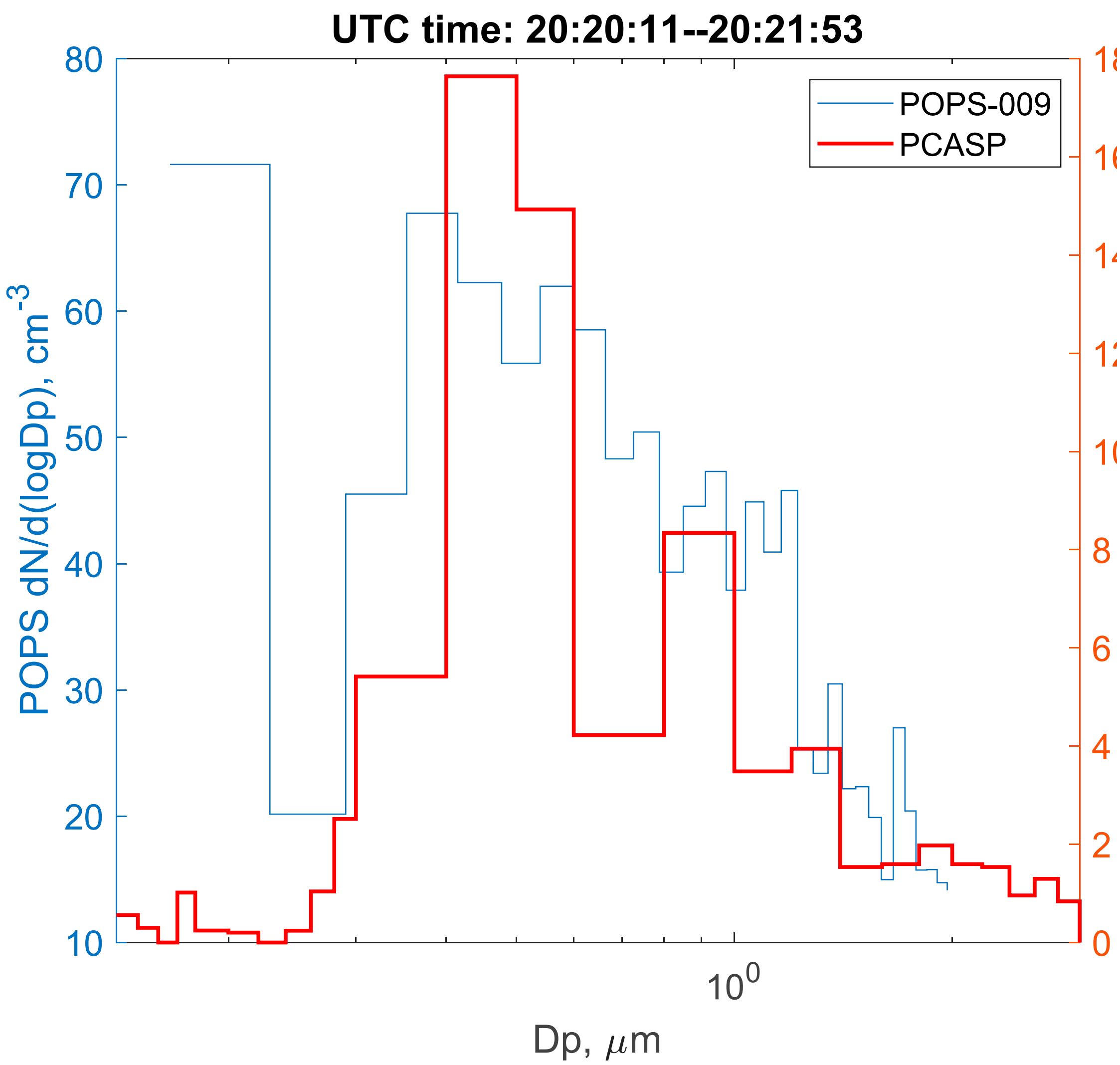
UTC time: 20:20:11--20:21:53
POPS-009
PCASP
POPS dN/d(logDp), cm-3
80
70
60
50
40
30
20
10
10^0
Dp, µm
0
2
4
6
8

(**a**)

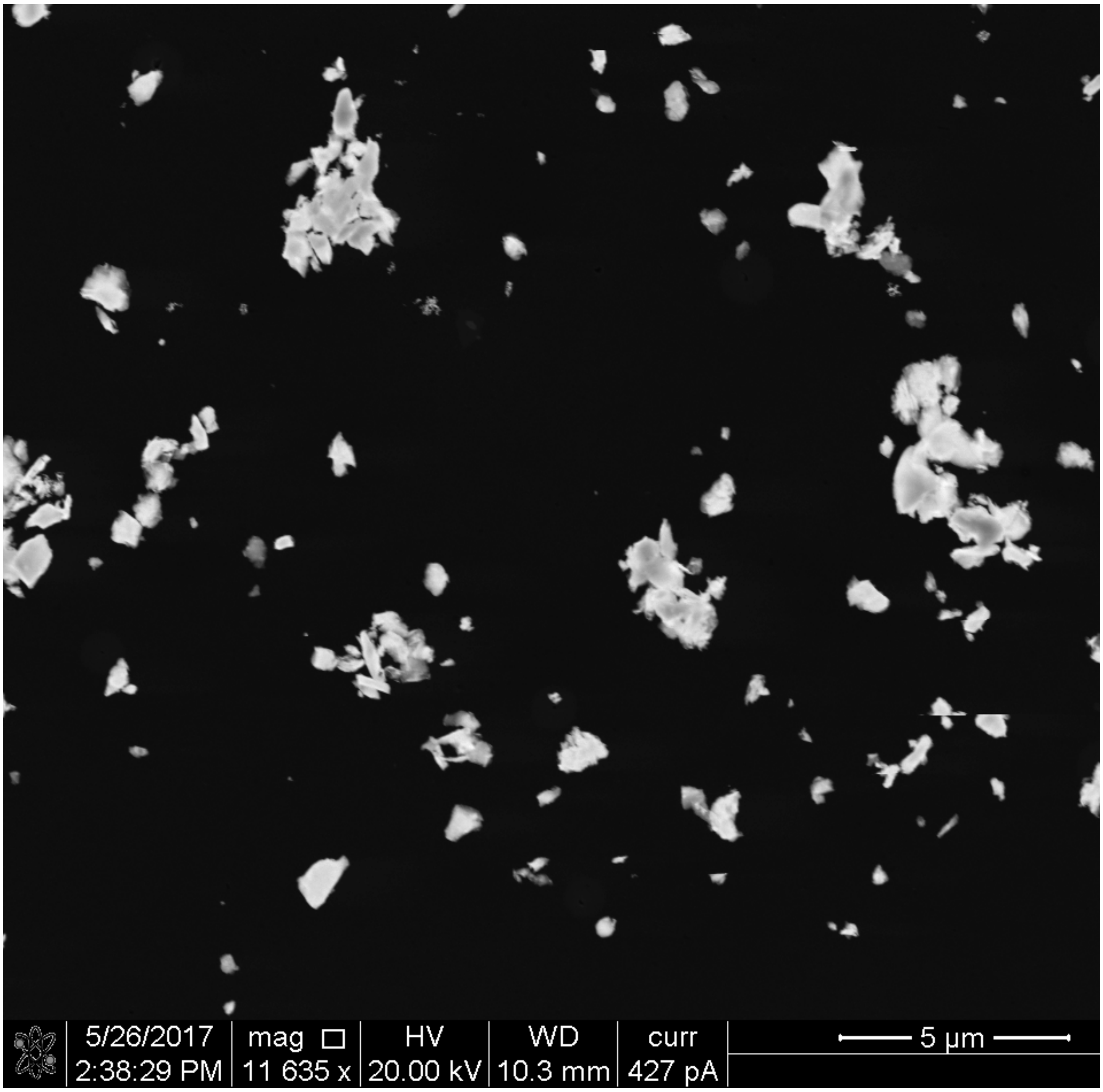

(**b**)

Figure S5. (**a**), POPS and PCASP size distribution of 500 nm (electrical mobility size) Arizona test dust (ATD) particles. (**b**), SEM images of Arizona test dust (ATD) particles.

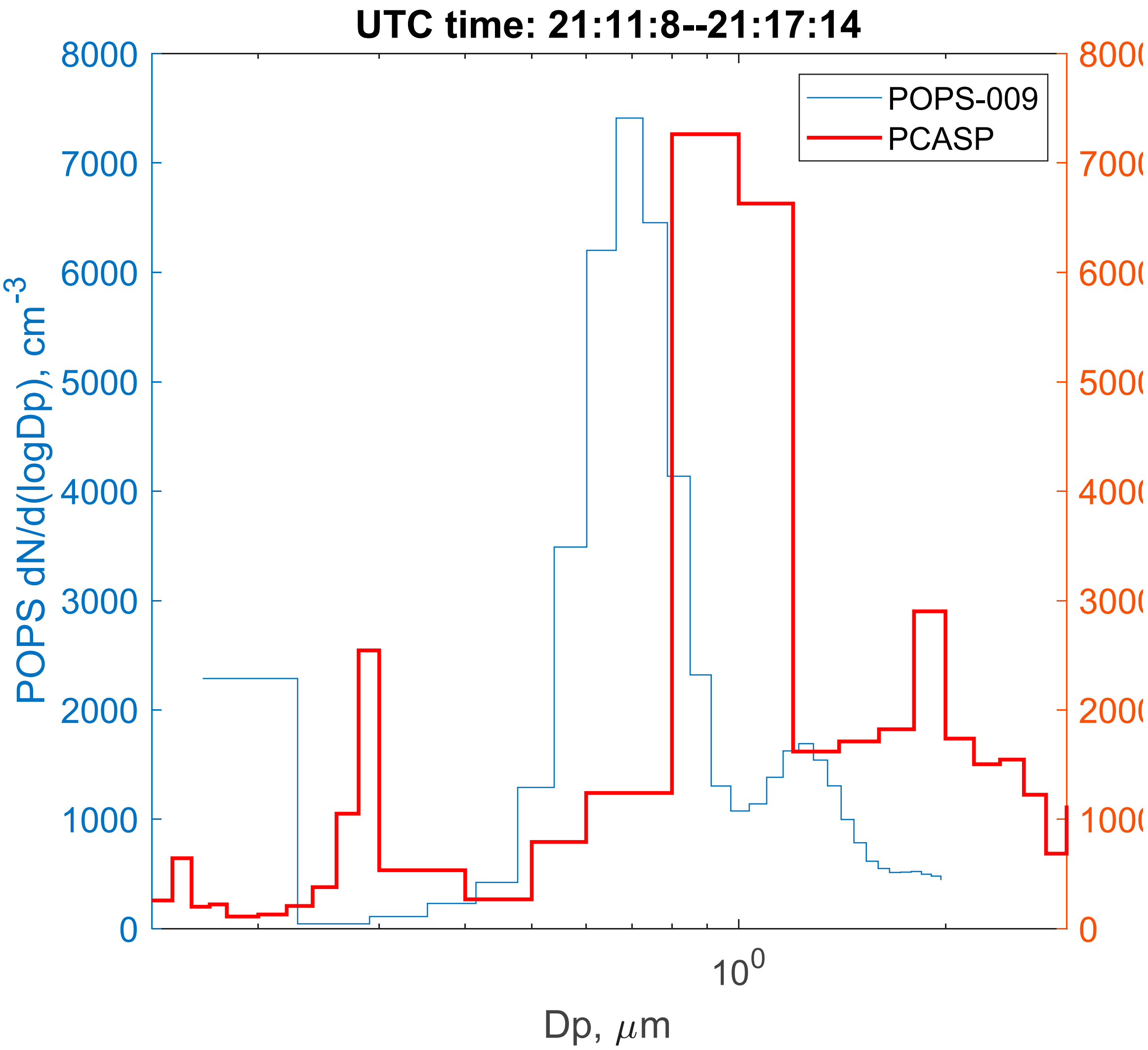
UTC time: 21:11:8--21:17:14
POPS-009
PCASP
POPS dN/d(logDp), cm-3
Dp, μm
10^0
0
1000
2000
3000
4000
5000
6000
7000
8000

(**a**)

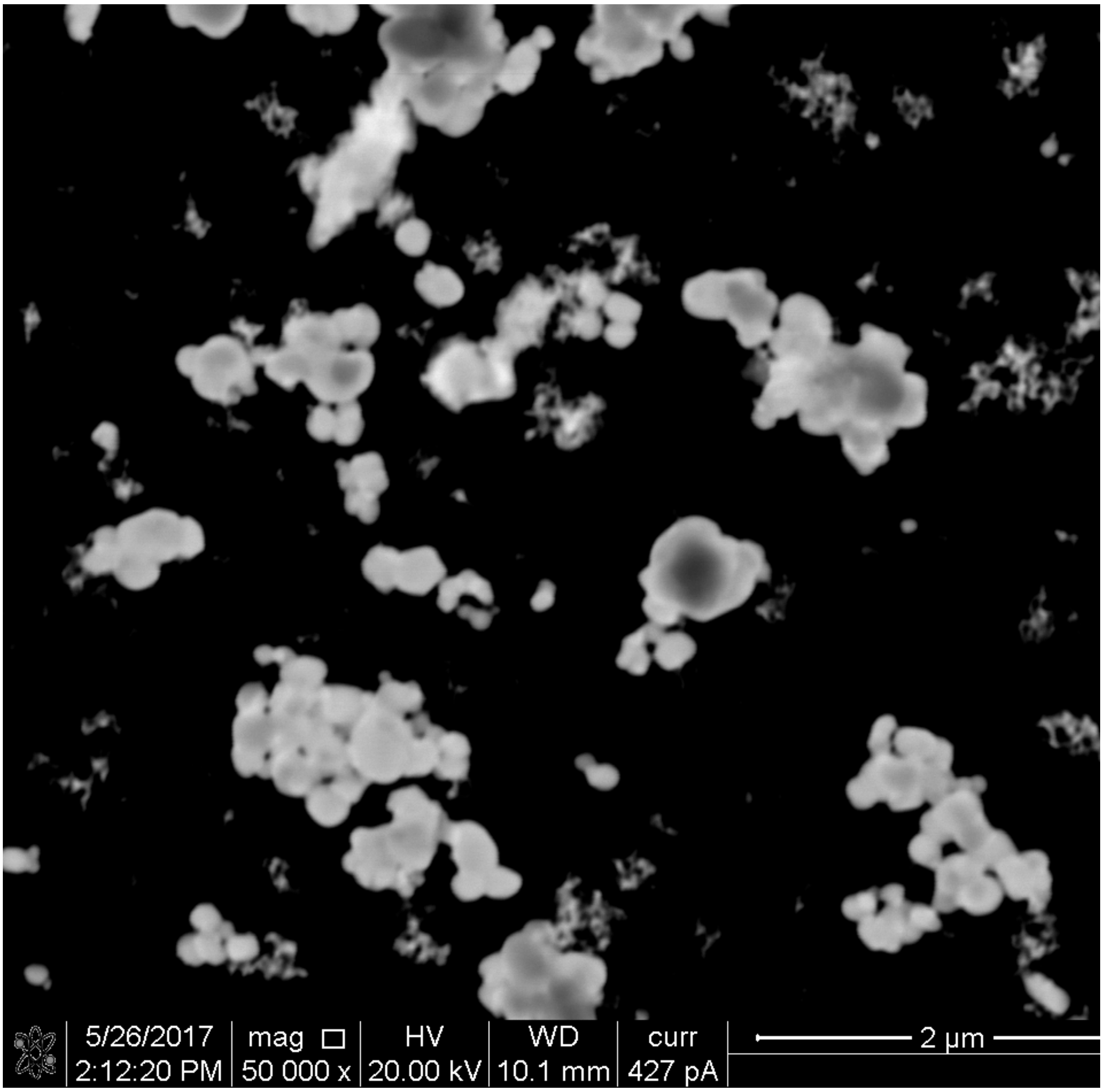

(**b**)

Figure S6. (**a**), POPS and PCASP size distribution of 500 nm (electrical mobility size) titanium dioxide particles. (**b**), SEM images of titanium dioxide particles.

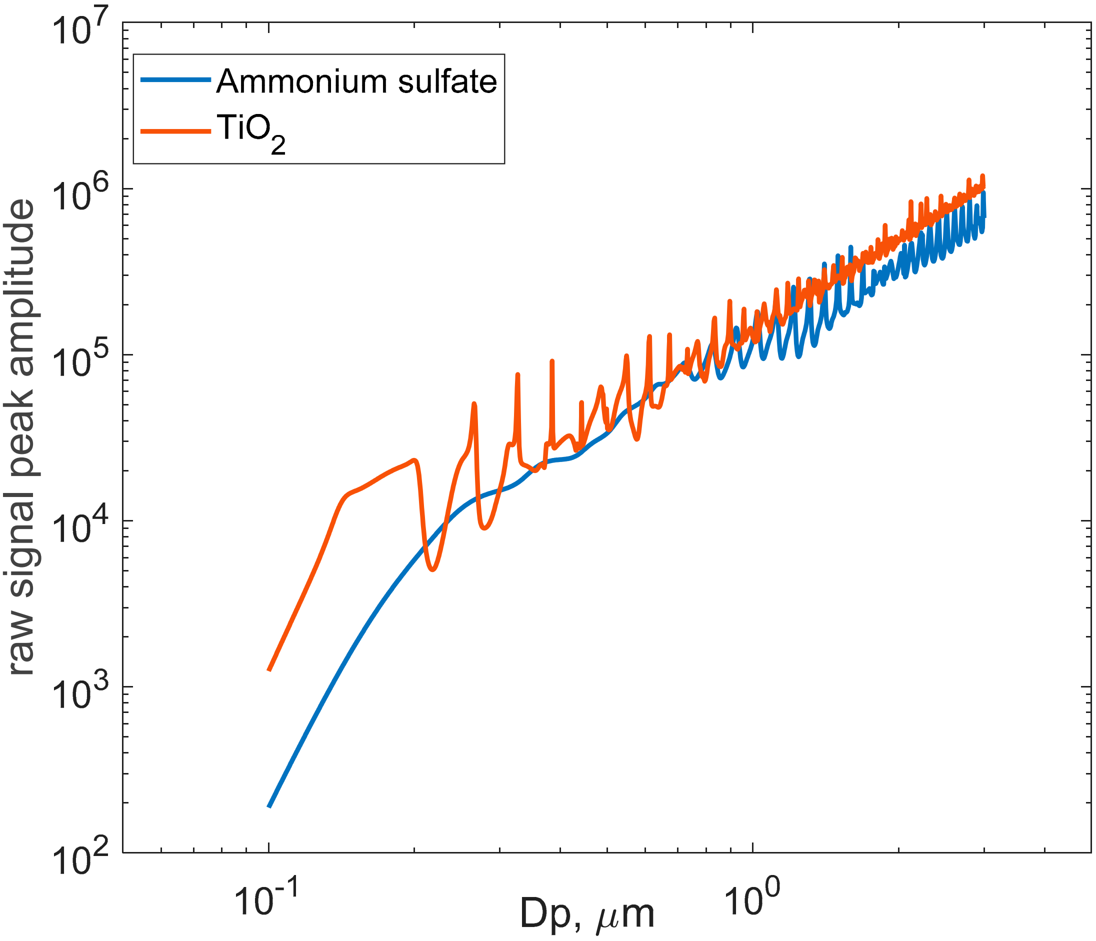
Ammonium sulfate
TiO2
raw signal peak amplitude
Dp, μm
10^7
10^6
10^5
10^4
10^3
10^2
10^-1
10^0

(**a**)

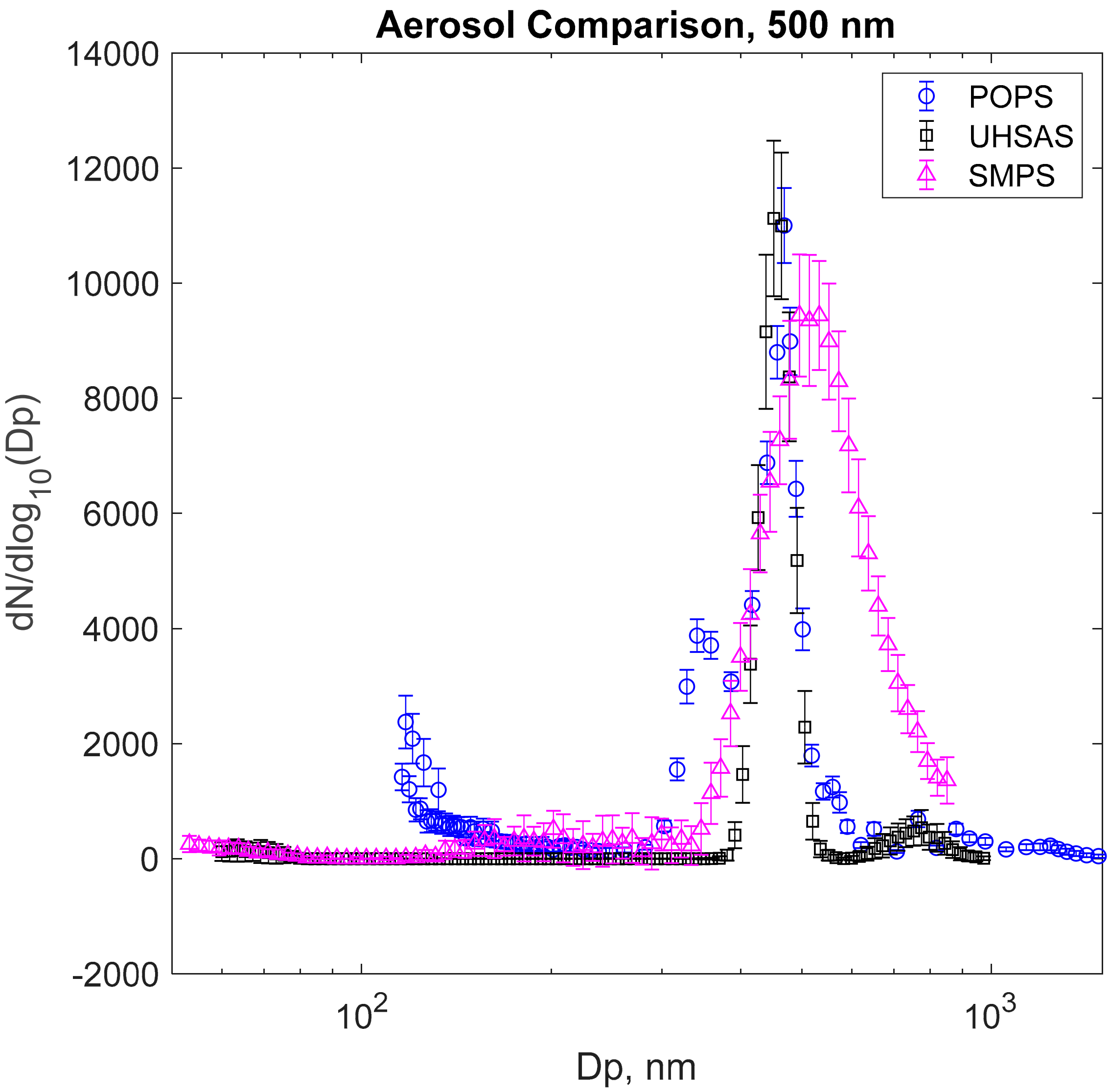

(**b**)

Figure S7. (**a**) Scattering amplitudes for Ammonium sulfate particles and TiO_2 particles calculated using Mie theory as a function of particle diameter. (**b**) Size distribution comparison of 500 nm (electrical mobility size) ammonium sulfate particles. With the POPS size bin number increased to 100 size bin, a small peak around 350 nm appeared only in the POPS size distribution.

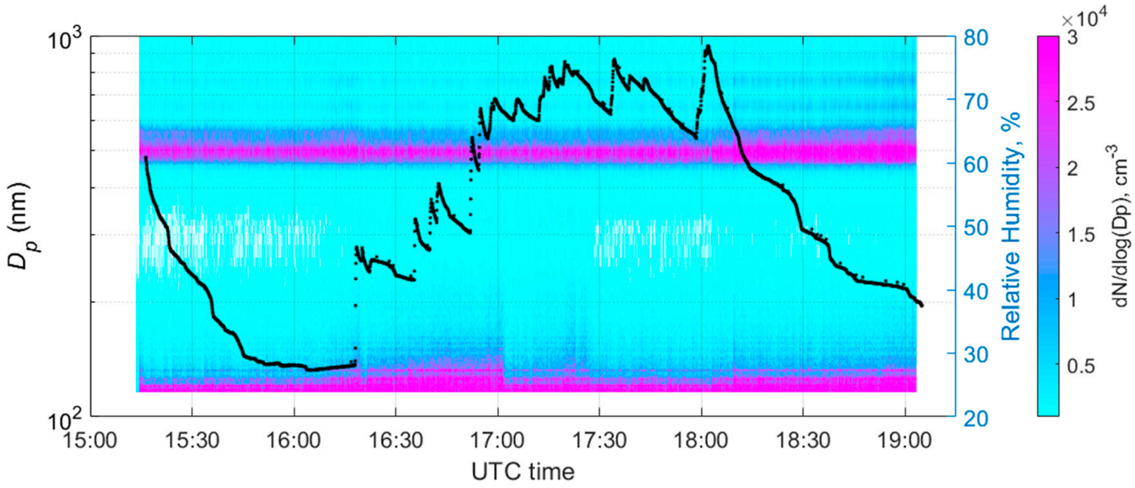

Figure S8. The relative humidity effect on the size distribution measurement for the 500 nm PSL aerosol particles.

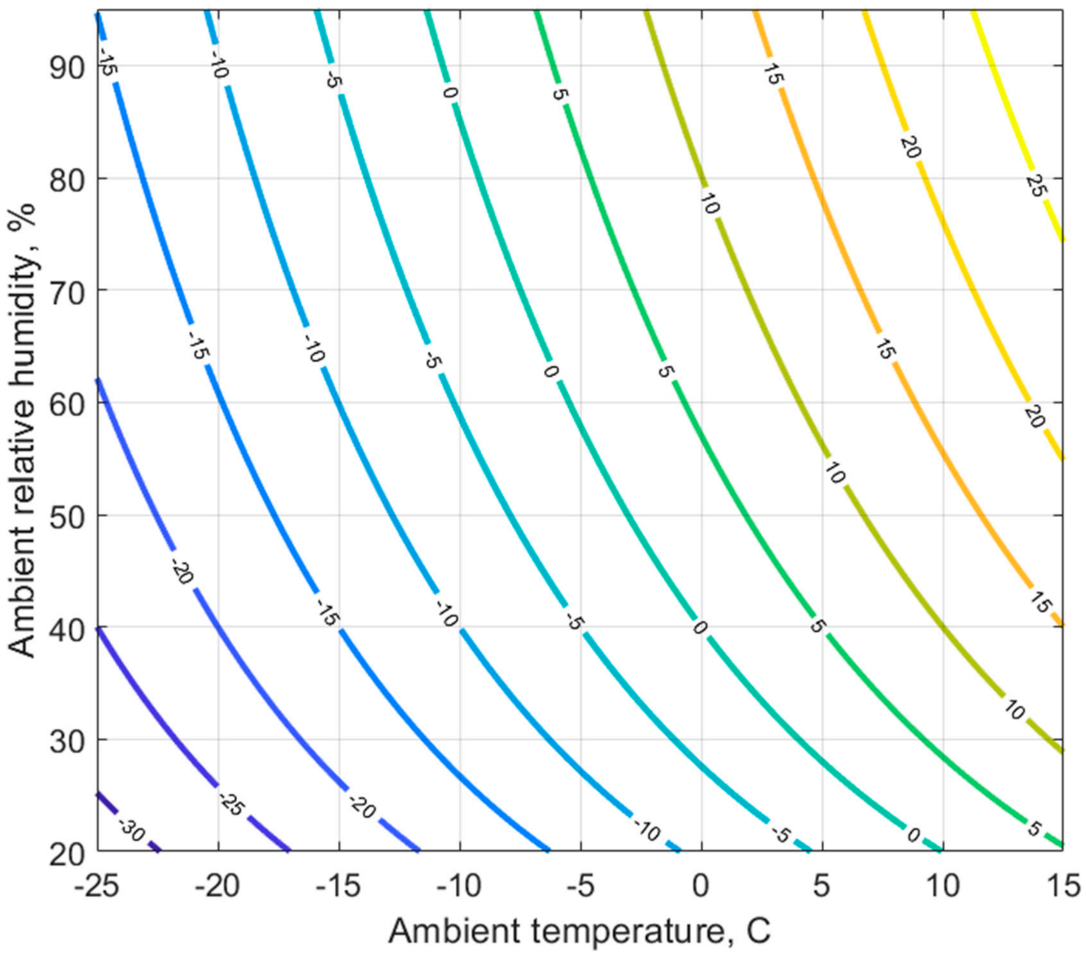

(**a**)

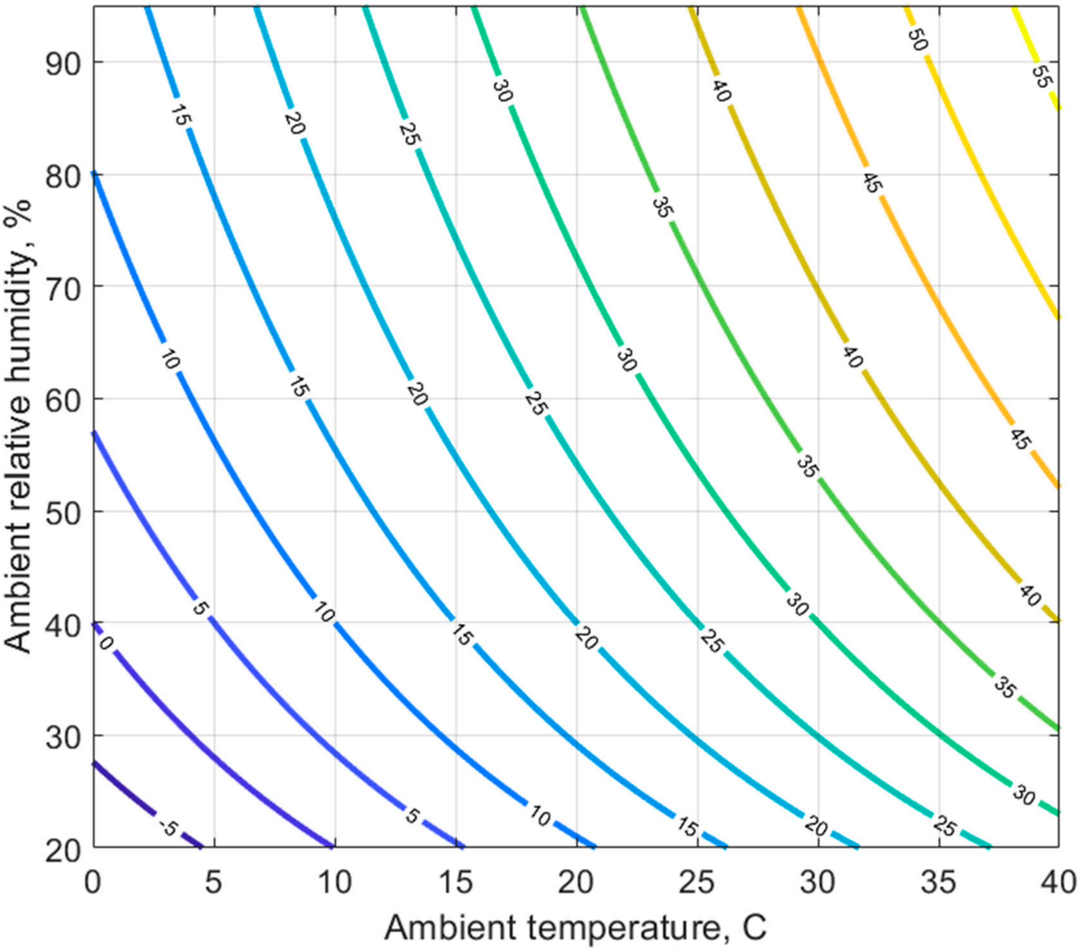

(**b**)

Figure S9. The estimated temperature difference between the ambient temperature and the POPS inlet\optical chamber, in order to achieve dry measuring conditions (i.e., RH = 40%) at variable ambient RH levels (**a**) for OLI site condition; (**b**) for SGP site condition. (based on the data between April and October 2018).

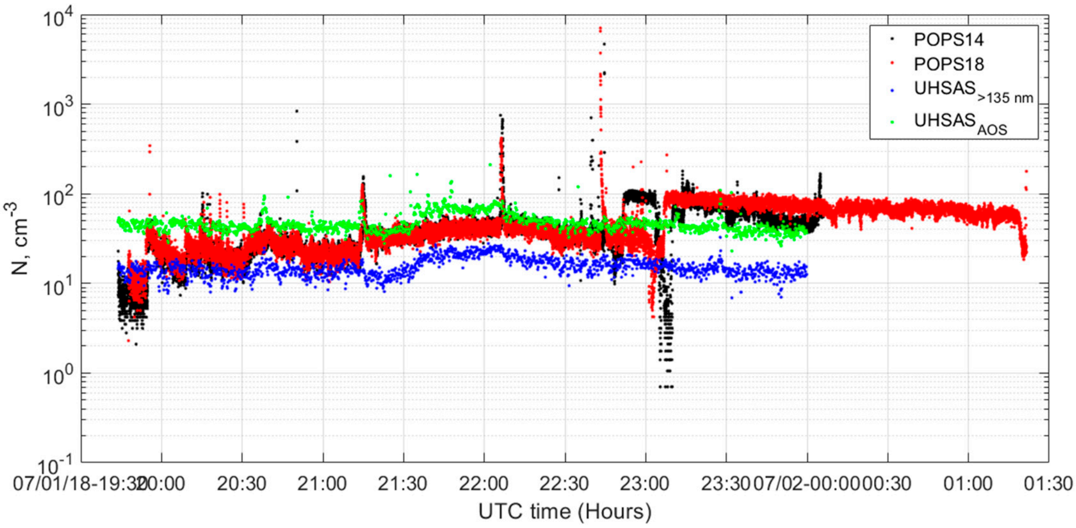

Figure S10. Comparison of total number concentration between two Tethered balloon system (TBS) POPS and the UHSAS from the ARM AMF3 during the POPEYE deployment in July 2018.

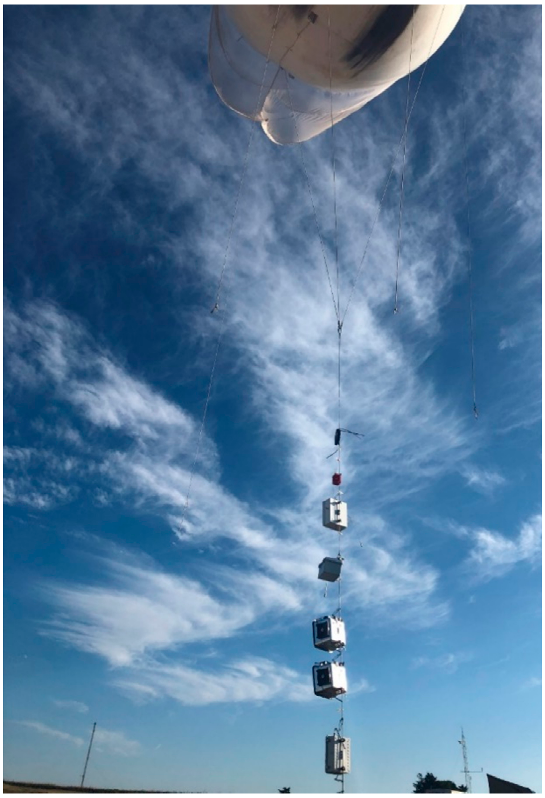

Figure S11. Tethered Balloon System (TBS) aerosol payload during the SGP (ARM site) deployment in July 2019.

Reference:

1. Wang, S. C.; Flagan, R. C. Scanning Electrical Mobility Spectrometer. *Aerosol Sci Tech* **1990,** *13*, 230–240.
2. Ardon-Dryer, K.; Garimella, S.; Huang, Y.-W.; Christopoulos, C.; Cziczo, D. Evaluation of DMA size selection of dry dispersed mineral dust particles. *Aerosol Sci Tech* **2015,** *49*, 828–841.
3. Curtis, D. B.; Meland, B.; Aycibin, M.; Arnold, N. P.; Grassian, V. H.; Young, M. A.; Kleiber, P. D. A laboratory investigation of light scattering from representative components of mineral dust aerosol at a wavelength of 550 nm. *J. Geophys. Res.: Atmos.* **2008,** *113*, D8.
4. DeCarlo, P. F.; Slowik, J. G.; Worsnop, D. R.; Davidovits, P.; Jimenez, J. L. Particle morphology and density characterization by combined mobility and aerodynamic diameter measurements. Part 1: Theory. *Aerosol Sci Tech* **2004,** *38*, 1185–1205.
5. Kulkarni, P.; Baron, P. A.; Willeke, K. *Aerosol measurement: principles, techniques, and applications*. John Wiley & Sons: city, country 2011.
6. Ku, B. K.; Kulkarni, P. Application of fractal theory to estimation of equivalent diameters of airborne carbon nanotube and nanofiber agglomerates. *Aerosol Sci Tech* **2018,** *52*, 597–608.

© 2020 by the authors. Submitted for possible open access publication under the terms and conditions of the Creative Commons Attribution (CC BY) license (http://creativecommons.org/licenses/by/4.0/).
